# Supplementary material for: Comprehensive Tools of Alkaloid/Volatile Compounds–Metabolomics and DNA Profiles: Bioassay-Role-Guided Differentiation Process of Six Annona sp. Grown in Egypt as Anticancer Therapy
Source: Pharmaceuticals (Basel). 2024 Jan 11;17(1):103. doi: 10.3390/ph17010103 (PMC10821326; doi:10.3390/ph17010103)
Supplement: Supplementary file 1 [file pharmaceuticals-17-00103-s001.zip › pharmaceuticals-2769255-supplementary.pdf]

Comprehensive Tools of Alkaloid/Volatile Compounds–Metabolomics and DNA Profiles: Bioassay-Role-Guided Differentiation Process of Six *Annona* sp. Grown in Egypt as Anticancer Therapy

Mona A. Mohammed [1,*], Nahla Elzefzafy [2], Manal F. El-Khadragy [3], Abdulhakeem Alzahrani [4], Hany Mohamed Yehia [4,5] and Piotr Kachlicki [6]

1 Medicinal and Aromatic Plants Research Department, Pharmaceutical and Drugs Industries Institute, National Research Centre, Dokki, Giza 12622, Egypt
2 Cancer Biology Department, National Cancer Institute, Cairo University, Cairo 11976, Egypt
3 Biology Department, Faculty of Science, Princess Nourah bint Abdulrahman University, P.O. Box 84428, Riyadh 11671, Saudi Arabia
4 Food Science and Nutrition Department, College of Food and Agricultural Sciences, King Saud University, P.O. Box 2460, Riyadh 11451, Saudi Arabia
5 Department of Food Science and Nutrition, Faculty of Home Economics, Helwan University, Helwan 11611, Egypt
6 Institute of Plant Genetics, Polish Academy of Sciences, 60-479 Poznan, Poland
* Correspondence: monaarafamohammed@yahoo.com or on.ibrahim@nrc.sci.eg

Table S1. Number of total bands, monomorphic bands and polymorphic bands and percentage of polymorphism revealed by the twelve 10-mer primers in the studied samples by SCoT and ISSR.

**SCoT**	**Primer Name**	**Total Band**	**Monomorphic Band**	**Polymorphic band**	**Unique Band**	**Polymorphic %**
	**SCoT 1**	8	4	4	-	50%
	**SCoT 2**	3	3	-	-	-
	**SCoT 3**	5	4	1	1	20%
	**SCoT 4**	7	3	4	4	57.14%
	**SCoT 6**	3	2	1	1	33.33%
	**SCoT 8**	5	1	4	3	80%
	**Total**	31	17	14	9	45.16%
**ISSR**	**14A**	4	3	1	-	25%
	**44A**	5	4	1	1	20%
	**HB-8**	5	3	2	-	40%
	**HB-10**	6	2	4	1	66.66%
	**HB-12**	8	5	3	3	37.5%
	**HB-13**	6	5	1	-	16.16%
	**Total**	34	22	12	5	35.29%
**Combination Between SCoT and ISSR**	ISSR	34	22	12	5	35.29%
	SCoT	31	17	14	9	45.16%
	Total	65	39	26	14	40.0%

Table S2. Similarity Index Using SCoT and ISSR analysis for six *Annona* sp.

species	1	2	3	4	5
1	1.0				
2	0.166	1.0			
3	0.233	0.538	1.0		
4	0.114	0.132	0.682	1.0	
5	0.589	0.238	0.875	0.530	1.0
6	0.308	0.283	0.845	0.413	0.378

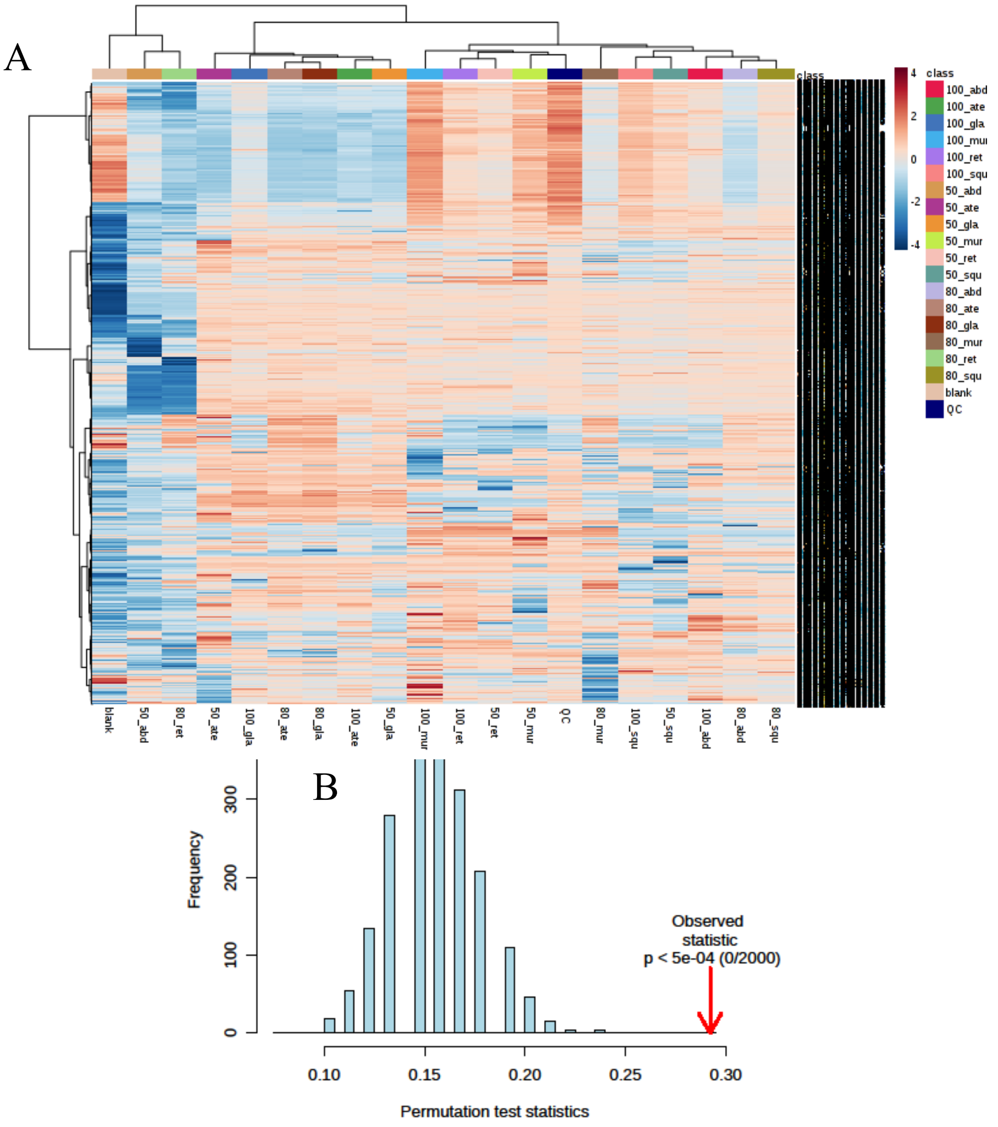

Figure S1. Bioinformatic pre-processing of LC/MS data resulted in detection of 67933 total signals in both modes in (A) the pearson correlation of heatmap of metabolic profiling of positive mode it apear different classification of compound showed in (sPLS-DA and PCA loading plot of different extract species. Hierachical clustering of all signals from different *Annona* species showed in clusters positive mode. (B) metabolomics data positive 18 samples with three replicates Empirical *p* value.

2. Taxonomic - DNA finger-printing for six *Annona* species grown in Egypt.

A. <u>SCoT analysis</u>

SCoT 1

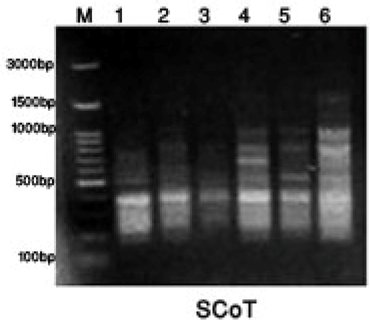

Band No	M.W bp	1	2	3	4	5	6
1	1740	0	0	0	1	0	1
2	1160	0	1	0	1	1	1
3	920	1	1	1	1	1	1
4	700	1	0	0	1	0	0
5	540	1	1	1	1	1	1
6	380	1	1	1	1	1	1
7	265	1	1	1	1	1	1
8	235	1	0	0	1	1	1
Total		6	5	4	8	6	7

SCoT 2

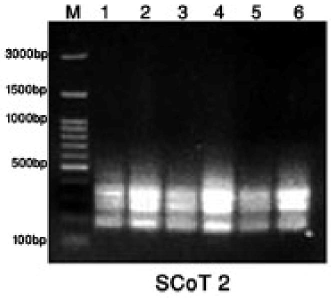

Band No	M.W bp	1	2	3	4	5	6
1	365	1	1	1	1	1	1
2	245	1	1	1	1	1	1
3	185	1	1	1	1	1	1
Total		3	3	3	3	3	3

SCoT 3

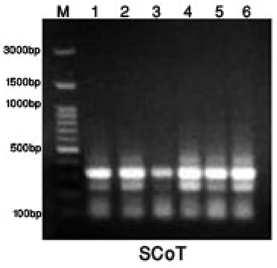

Band No	M.W bp	1	2	3	4	5	6
1	400	1	1	0	1	1	1
2	330	1	1	1	1	1	1
3	245	1	1	1	1	1	1
4	175	1	1	1	1	1	1
5	130	1	1	1	1	1	1
Total		5	5	4	5	5	5

SCoT 4

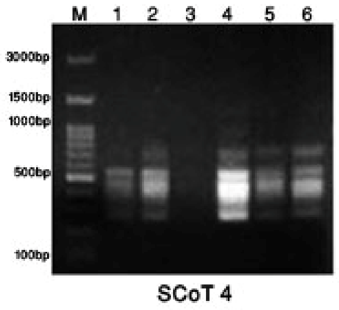

Band No	M.W bp	1	2	3	4	5	6
1	1185	0	0	0	1	0	0
2	840	0	0	0	1	0	0
3	715	1	1	0	1	1	1
4	550	1	1	1	1	1	1
5	470	1	1	1	1	1	1
6	380	1	1	1	1	1	1
7	260	1	1	0	1	1	1
Total		5	5	3	7	5	5

SCoT 6

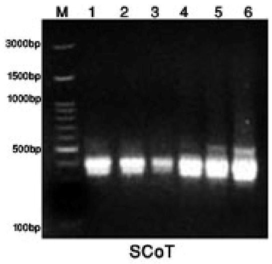

Band No	M.W bp						
		1	2	3	4	5	6
1	565	1	1	0	1	1	1
2	400	1	1	1	1	1	1
3	345	1	1	1	1	1	1
Total		3	3	2	3	3	3

SCoT 8

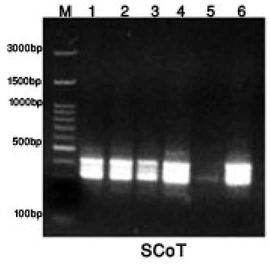

Band No	M.W bp						
		1	2	3	4	5	6
1	500	1	1	0	1	0	1
2	430	1	1	1	1	0	1
3	370	1	1	1	1	0	1
4	315	1	1	1	1	1	1
5	245	1	1	1	1	0	1
Total		5	5	4	5	1	5

Figure S2. DNA of primers in the studied 6 *Annona* samples by SCoT. As 1 = *A. atemoya*, 2 = *glabra*, 3 = *abdel razek,* 4 = *reticulata,* 5 = *squamosa*, then 6 = *muricata.*

B. ISSR analysis

The ISSR banding profiles produced by the six 10-mer primers in the six samples of *Annona* species are illustrated in Fig.(5) and Tables 5for primers14A, 44B, HB-08,HB-10, HB-12 and HB-14.

14A

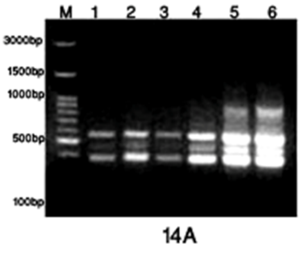

Band No	M.W bp	Samples					
		1	2	3	4	5	6
1	835	0	0	0	0	1	1
2	580	1	1	1	1	1	1
3	475	1	1	1	1	1	1
4	390	1	1	1	1	1	1
Total		3	3	3	3	4	4

44A

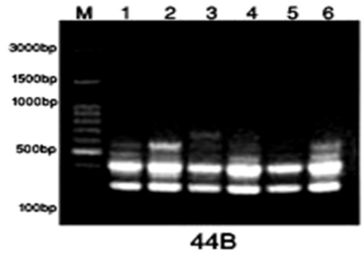

Band No	M.W bp	Samples					
		1	2	3	4	5	6
1	700	0	0	1	0	0	0
2	580	1	1	1	1	1	1
3	500	1	1	1	1	1	1
4	400	1	1	1	1	1	1
5	270	1	1	1	1	1	1
Total		4	4	5	4	4	4

HB-8

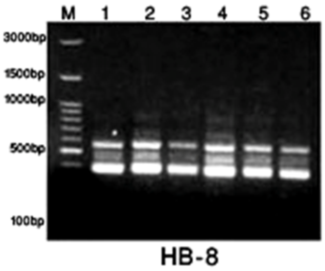

Band No	M.W bp	Samples					
		1	2	3	4	5	6
1	845	0	1	0	1	1	0
2	715	0	1	0	1	1	0
3	580	1	1	1	1	1	1
4	485	1	1	1	1	1	1
5	400	1	1	1	1	1	1
Total		3	5	3	5	5	3

HB-10

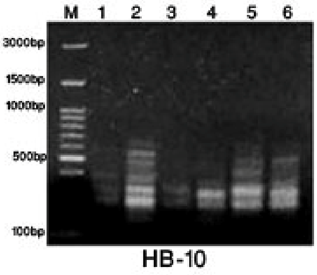

Band No	M.W bp	Samples					
		1	2	3	4	5	6
1	650	0	1	0	0	1	1
2	570	0	1	0	0	0	0
3	480	0	1	0	0	1	1
4	430	0	0	0	0	1	0
5	300	1	1	1	1	1	1
6	265	1	1	1	1	1	1
Total		2	5	2	2	5	4

HB-12

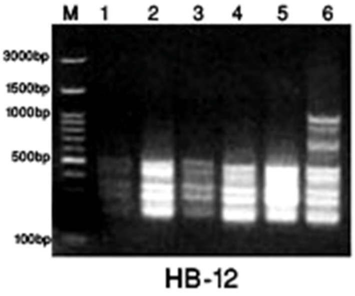

Band No	M.W bp	Samples					
		1	2	3	4	5	6
1	1040	0	0	0	0	0	1
2	900	0	0	0	0	0	1
3	680	0	0	0	0	0	1
4	510	1	1	1	1	1	1
5	425	1	1	1	1	1	1
6	365	1	1	1	1	1	1
7	270	1	1	1	1	1	1
8	230	1	1	1	1	1	1
Total		6	6	6	6	6	6

HB-13

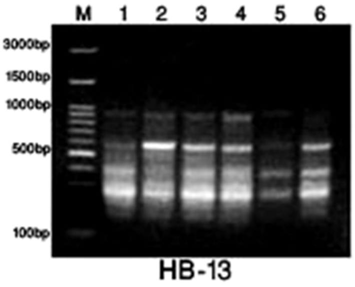

Band No	M.W bp	Samples					
		1	2	3	4	5	6
1	910	1	1	1	1	1	1
2	600	1	1	1	1	1	1
3	480	1	1	1	1	0	0
4	400	1	1	1	1	1	1
5	290	1	1	1	1	1	1
6	180	1	1	1	1	1	1
Total		6	6	6	6	5	5

Figure S3. DNA of primers in the studied 6 Annona samples by ISSR. As 1 = *A. atemoya*, 2 = *glabra*, 3 = *abdel razek,* 4 = *reticulata,* 5 = *squamosa*, then 6 = *muricata.*

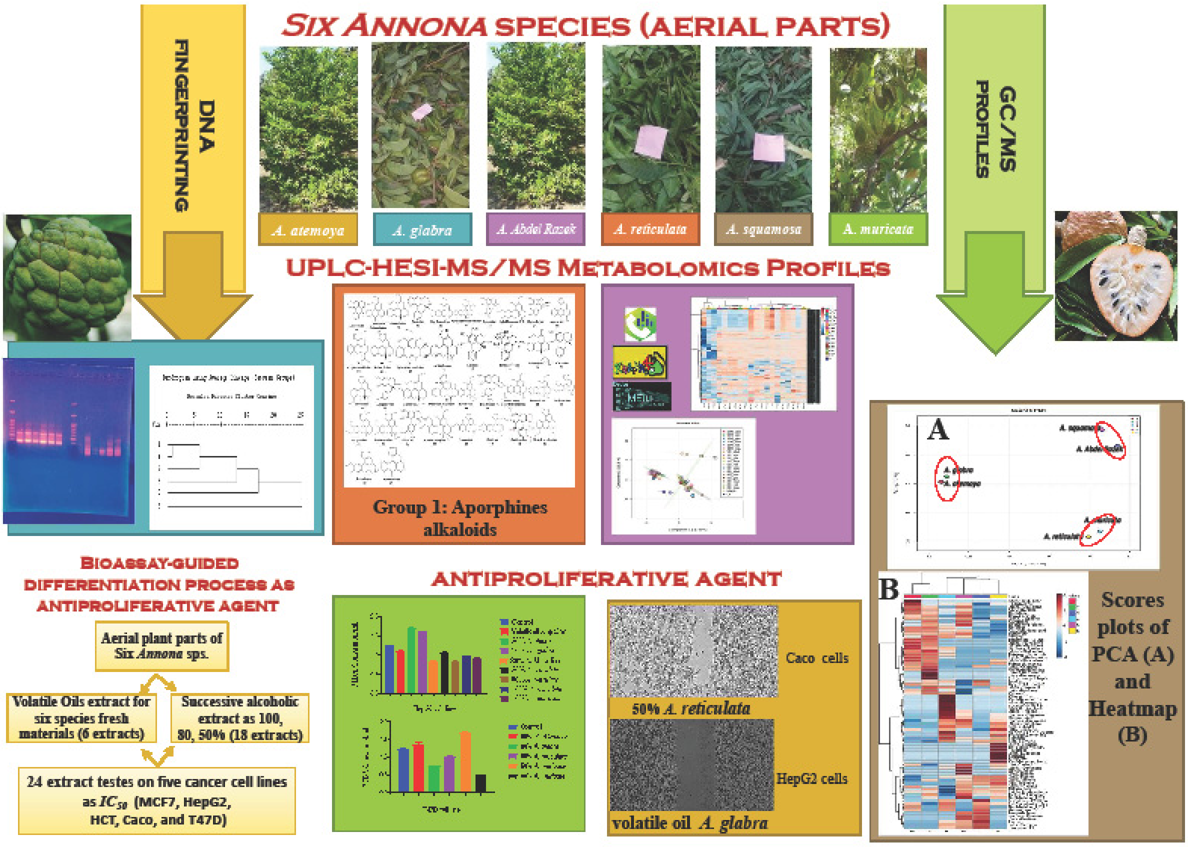

Figure S4. Role-bioassay-guided differentiation process of Six *Annona* cultivated in Egypt on anti-cancer therapy.
